# The “Old” and the “New” Antibiotics for MDR Gram-Negative Pathogens: For Whom, When, and How

**DOI:** 10.3389/fpubh.2019.00151

**Published:** 2019-06-11

**Authors:** Ilias Karaiskos, Styliani Lagou, Konstantinos Pontikis, Vasiliki Rapti, Garyphallia Poulakou

**Affiliations:** ^1^First Department of Internal Medicine-Infectious Diseases, Hygeia General Hospital, Athens, Greece; ^2^Third Department of Medicine, School of Medicine, Sotiria General Hospital, National and Kapodistrian University of Athens, Athens, Greece; ^3^ICU First Department of Respiratory Medicine, School of Medicine, Sotiria General Hospital, National and Kapodistrian University of Athens, Athens, Greece

**Keywords:** ceftazidime avibactam, ceftolozane tazobactam, colistin, combination, monotherapy, carbapenemase producing *Klebsiella pneumoniae*, *Acinetobacter baumannii*, *Pseudomonas aeruginosa*

## Abstract

The recent expansion of multidrug resistant and pan-drug-resistant pathogens poses significant challenges in the treatment of healthcare associated infections. An important advancement, is a handful of recently launched new antibiotics targeting some of the current most problematic Gram-negative pathogens, namely carbapenem-producing Enterobacteriaceae (CRE) and carbapenem-resistant *P. aeruginosa* (CRPA). Less options are available against carbapenem-resistant *Acinetobacter baumannii* (CRAB) and strains producing metallo-beta lactamases (MBL). Ceftazidime-avibactam signaled a turning point in the treatment of KPC and partly OXA- type carbapenemases, whereas meropenem-vaborbactam was added as a potent combination against KPC-producers. Ceftolozane-tazobactam could be seen as an ideal beta-lactam backbone for the treatment of CRPA. Plazomicin, an aminoglycoside with better pharmacokinetics and less toxicity compared to other class members, will cover important proportions of multi-drug resistant pathogens. Eravacycline holds promise in the treatment of infections by CRAB, with a broad spectrum of activity similar to tigecycline, and improved pharmacokinetics. Novel drugs and combinations are not to be considered “panacea” for the ongoing crisis in the therapy of XDR Gram-negative bacteria and colistin will continue to be considered as a fundamental companion drug for the treatment of carbapenem-resistant Enterobacteriaceae (particularly in areas where MBL predominate), for the treatment of CRPA (in many cases being the only *in vitro* active drug) as well as CRAB. Aminoglycosides are still important companion antibiotics. Finally, fosfomycin as part of combination treatment for CRE infections and *P. aeruginosa*, deserves a greater attention. Optimal conditions for monotherapy and the “when and how” of combination treatments integrating the novel agents will be discussed.

## Introduction

Treatment and outcomes of healthcare-acquired infections have been threatened by the worldwide increasing incidence of antimicrobial resistance among Gram-negative bacteria (1). New terminology was launched to describe extensively drug-resistant (XDR) and pan drug-resistant (PDR) Gram-negative microorganisms (2), with very limited treatment options and major representatives *Klebsiella pneumoniae, Acinetobacter baumannii*, and *Pseudomonas aeruginosa* (1, 3). Several official reports including those of the World Health Organization (WHO), the Infectious Diseases Society of America (IDSA) and the UK Government in 2015 have designated antimicrobial resistance as one of the major problems affecting human health and health economy (4–6). As a result of the pressure exerted from regulatory bodies and social waves, we have a handful of already launched new antibiotics targeting some of the current most problematic Gram-negative pathogens, namely *Klebsiella pneumoniae* carbapenemase (KPC)- producing Enterobacteriaceae and multi-drug-resistant (MDR) *P. aeruginosa*. There are more new antimicrobials in the final stage of development, some of them targeting pathogens expressing metallo-beta lactamases, MDR *A. baumannii* and other problematic pathogens, whereas novel non-antibiotic approaches are in development, in order to confront pathogens' ability to develop resistance to new antibiotic classes (7–9). Currently, ceftazidime-avibactam, ceftolozane-tazobactam, meropenem-vaborbactam, and eravacycline have been added to our armamentarium in US and Europe, whereas plazomicin has already got Food and Drug Adminstration (FDA) approval. For all recently launched antimicrobials, evidence about the optimal use outside registration trials is accumulating, though not clear yet. Unmet clinical needs may be geographically diverse and different from regulatory-approved indications (10). Furthermore, lessons learned from the previous antibiotic dawn, call for vigilance for emergence of resistance in every new antibiotic. Prudent use of these precious additions in our antimicrobial armamentarium is important to ensure their longevity (1). In this narrative review, first we will appraise old- revived (colistin, fosfomycin) or established (tigecycline-aminoglycosides-carbapenems) antibiotics that are being used in the treatment of XDR pathogens, with focus on their drawbacks as optimal treatment options and their potential as treatment components along with new antibiotics. Then we will present a summary of new antibiotics centered in real-life use and try to define the most appropriate candidate-patient to whom they must be prescribed. Finally, the “when and how” of combination treatments will be appraised.

## Prevalent Mechanisms of Resistance Among Difficult-to-treat Pathogens

New drugs are called to encounter pathogens with accumulating mechanisms of resistance. Carbapenem resistance is a pivotal event in the generation of XDR pathogens, because a potent antibiotic class is inactivated, along with all beta-lactams, when the underlying mechanism is the production of a carbapenemase, which acts as a broad beta-lactamase (11). Clinical implications are profound, due to the exhaustion of therapeutic options. Enterobacteriaceae, with *Klebsiella pneumoniae* the most representative, *P. aeruginosa* and *A. baumannii* are the most common pathogens in clinical practice that harbor carbapenemases. Table 1 shows the most relevant mechanisms of resistance encountered among XDR and PDR isolates (11–14). The majority of acquired carbapenemases belong to either Ambler class A, or class B (metallo-beta-lactamases–MBLs), or class D (oxacillinases–OXAs) (13). Extended-spectrum beta-lactamases (ESBLs), although not conferring resistance to carbapenems, are important contributors of resistance traits because ESBL-, and carbapenemsae- encoding plasmids are frequently vectors of resistance determinants for other antimicrobial classes, such as aminoglycosides (aminoglycoside-modifying enzymes or 16S rRNA methylases) and fluoroquinolones (Qnr, or efflux pumps) (11–13).

**Table 1:** Prevalent mechanisms of resistance among pathogens with extended-drug resistance (XDR) (11–14).

**Classification**	**Mechanism**	**Common bacterial species**	**Examples**	**Substrate**
B-lactamase Ambler class A	Extended-spectrum Or ESBLs	Enterobacteriaceae, Pseudomonas aeruginosa, Acinetobacter spp., Kluyvera spp.	SHV-like, CTX-like, KLUG-like	Penicillins, cephalosporins (except cefamycins), aztreonam Frequently co-transferred with VIM
B-lactamase Ambler class A	Serine carbapenemases Acquisition of a mobile genetic element	*Klebsiella spp*.	KPC-like, IMI-like	Penicillins, cephalosporins, aztreonam, carbapenems
B-lactamase Ambler class B	Metallo-β-lactamases, carbapenemases Acquisition of a mobile genetic element	*Stenotrophomonas maltophilia, P. aeruginosa, Bacteroides fragilis, Acinetobacter baumannii*	VIM-like, IMP-like, NDM-like, GIM, SPM, SIM	Penicillins, cephalosporins, and carbapenems. Monobactams are stable
B-lactamase Ambler class C	Extended-spectrum, cephalosporinases, Mainly Chromosomal	*Enterobacter spp., Klebsiella spp., Proteus spp., Citrobacter spp., E. coli*	AmpC, P99, ACT-like, CMY-like, MIR-like	–
B-lactamase Ambler class D	Carbapenemases	*A. baumannii, P. aeruginosa, E. coli*,	OXA-like (OXA-51, OXA-23)	Penicillin, aztreonam, and carbapenems
Porin mutations (Loss of outer membrane permeability)	Chromosomal mutation	*P.aeruginosa*, *A. baumannii*	OprD CarO,	Imipenem
Efflux pumps	Chromosomal mutations Different antimicrobial classes may be substrates of a single pump: exposure to a given class (e.g., beta-lactams) may thereby select mutants with resistance to other classes	*P. aeruginosa*	MexAB-OprM	Ticarcillin, aztreonam, cefepime, meropenem, quinolones
–	–	*A. baumannii*	AdeABC	Beta-lactams (variable), aminoglycosides, fluoroquinolones, tigecycline
Topoisomerase modifications Gyrase modifications	Chromosomal mutation	*P.aeruginosa, A. Baumannii*, Enterobacteriaceae	–	Fluoroquinolones
Qnr	Plasmid mediated	Enterobacteriaceae	A, B, C, D and S subtypes	Fluoroquinolones
Aminoglycoside-modifying enzymes	Acquisition of a mobile genetic element Aminoglycoside phosphotransferase, APH, aminoglycoside nucleotidyltransferase, ANT and aminoglycoside acetyltransferase, AAC	Enterobacteriaceae, *A. baumannii*	AAC(3), AAC(6') and APH(3′)	Aminoglycosides
Methylases of the 16S ribosomal subunit	Acquisition of a mobile genetic element	NDM-1 producing strains (mainly Enterobacteriaceae)	ArmA Rmt	Plazomicin is stable against the majority of AMEs but is being hydrolysed by Rmts
Lipid A (LPS) modifications	Chromosomal mutation	*P.aeruginosa, K. Pneumoniae, A. baumannii*	–	Colistin
PmrA–PmrB two-component system genetic modifications	Chromosomal mutation	*A. baumannii, P.aeruginosa, K. Pneumoniae*	–	Colistin
Mcr1 gene mutation	Plasmid mediated	Enterobacteriaceae	–	Colistin

Outer membrane modifications and particularly mutations in membrane porins may confer resistance to carbapenems without resistance to other beta-lactams. Efflux pumps confer resistance to carbapenems without carbapenemase production; they may affect multiple antibiotics. Aminoglycoside-modifying enzymes confer resistance to aminoglycoside class; usually they are located in mobile genetic elements along with other mechanisms of resistance (11–13). Finally resistance to colistin was chromosomally mediated for years; a plasmid-mediated mechanism through mutation in mcr1 gene is a new threat, first identified from Enterobacteriaceae in China (14).

## Old and Established Antibiotics

### Colistin

Colistin, systemically administered in the form of the prodrug colistin methanesulfonate (CMS), is a revived antibiotic that has been addressed as the last resort antibiotic of the twenty-first century due to the steep increase of resistance rates and the lack of new effective antimicrobial agents (15). The antimicrobial spectrum of colistin includes MDR and XDR Gram-negatives regardless of mechanism of resistance, mainly *K. pneumoniae, A. baumannii*, and *P. aeruginosa*, whereas Proteae are inherently resistant (15). *In vitro* activities of colistin and novel combination ceftazidime/avibactam (CAZ-AVI) against isolates of Enterobacteriaceae collected in Europe as part of the International Network For Optimal Resistance Monitoring (INFORM) global surveillance program from 2012 to 2015 revealed CAZ-AVI as the most potent active agent with a susceptibility rate of 99.4%, whereas susceptibility to colistin was 82.8% (16). Regarding *P. aeruginosa*, colistin was the most active drug followed by CAZ-AVI with a susceptibility rate of 99.6 and 92.4%, respectively (17).

During the last decade, a better characterization of the colistin pharmacokinetics (PKs) has been made feasible due to advanced technology, resulting in minimization of hydrolysis of CMS to colistin during workup, introducing colistin into a revolutionary period (15). The necessity of a loading dose and longer dosing intervals (18, 19) as well as a dosing scheme based on creatinine clearance (CrCL) have been proposed (20) and assimilated in clinical practice (19–21). The updated colistin daily maintenance doses have been shown to achieve target attainment of clinically relevant plasma colistin concentrations (around 2 mg/L) for approximately 90% for patients with CrCl < 80 ml/min (22). However, the recent observation that a high CrCL of >80 mL/min decreases the ability to achieving the appropriate steady-state colistin levels attributed to a higher amount of colistin cleared by the kidneys, probably mandates either the necessity of combinations or higher dosing (5, 8, 19, 22).

The current clinical efficacy of colistin is extrapolated from reported studies focusing mainly on bacteremias and VAP caused by MDR and XDR Gram-negative pathogens. However, the major drawbacks of publications are their retrospective, non-randomized design, with a variety of dosages, and the absence of a loading dose, whereas the simultaneous administration of other active *in vitro* antibiotics renders inconclusive the efficacy of monotherapy with colistin (15). The issue of combination therapy with colistin has received considerable critical attention and is a debated and controversial topic (23, 24). Regarding carbapenem resistant Enterobacteriaceae (CRE), the combination of colistin with another active *in vitro* antibiotic has been reported to be beneficial in terms of survival, as depicted in a big number of patients and despite their retrospective source of the studies (23–25). The combination of meropenem (if MIC is ≤8 mg/L) with gentamicin or tigecycline or colistin have resulted significantly in reduction of mortality particularly in patients with septic shock, high mortality score, or rapidly fatal underlying diseases (25, 26). However, in low-risk bloodstream infections (BSIs) and non-bacteremic urinary and abdominal infections, monotherapy seems as an adequate therapeutic choice (25). There are currently minimal and of low-quality clinical evidence suggesting superiority of monotherapy over combination therapy for treatment of carbapenem resistant *P. aeruginosa* (CRPA). However, many clinicians, taking into consideration PK/PD limitations of colistin and the development of resistances favor combination treatment (23, 24). Similarly, colistin has been frequently used in combination for the treatment of carbapenem resistant *A. baumannii* (CRAB), with the same concerns regarding the suboptimal PK of polymyxins (23, 24). This concept has recently been challenged by randomized control trials focusing on infections caused by CRAB demonstrating superior efficacy of monotherapy of colistin vs. combination therapy (27–29). The most recent study enrolled 151 patients with CRAB infections treated with colistin alone vs. colistin plus meropenem and rates of clinical failure (83 vs. 81%, *p* = 0.64) and mortality (46 vs. 52%, *p* = 0.40) were found similar in both groups. However, the unexpected high rates of clinical failure and mortality, highlights the necessity of novel agents to effectively treat CRAB infections (29).

### Fosfomycin

Having been discovered in the ‘60s, fosfomycin in its intravenous form, fosfomycin disodium (FD), was soon almost abandoned due to the availability of other agents and the peculiarities of susceptibility testing (30). It was not until about a decade ago that it was reintroduced into clinical practice, with a new role in the ongoing fight against XDR and PDR Gram-negative bacteria in critically ill patients (30, 31).

Fosfomycin exerts bactericidal action against susceptible organisms and possesses a unique mechanism of inhibiting the first step of peptidoglycan synthesis, making cross-resistance with other agents unlikely (32). FD has straightforward pharmacokinetics, though less well studied in the critically ill. High concentrations are achieved in serum and urine, well-above minimum inhibitory concentrations (MICs) of susceptible organisms, while penetration to compartments relevant to the ICU setting (lung, cerebrospinal fluid, abscess fluid) is satisfactory (33–35). FD has very few toxicity concerns (36, 37). Hypokalemia and sodium overload stand out as the most important; however, they are usually controlled in the ICU setting.

In contemporary *in vitro* studies fosfomycin was active against more than 80% of *Staphylococcus aureus, Enterococcus faecium*, ESBL-producing *Escherichia coli*, and *K. pneumoniae* and slightly less against carbapenem-resistant (CR) *K. pneumoniae* (38). Less information was available for P*. aeruginosa*, but at least in one study, 80.6% of strains were inhibited by 128 mg/L of fosfomycin (39). Against a recent collection of 396 *K. pneumoniae* isolates originating from Greek hospitals, fosfomycin was the third most active agent, being active against 58% of strains by using the strict EUCAST criteria for *E. coli* (where susceptibility break point is 32 mg/L as opposed to 64 mg/L for CLSI) (40). These results were confirmed in a recent collection of US bacterial isolates (41). Of great interest to intensivists is the absence of activity against *A. baumannii* strains (30). The *in vivo* development of resistance during treatment is the most feared complication of FD use (41) driving repeated recommendations for use only within combination regimens (8, 30). Recent reports however even with monotherapy did not verify this fear (37).

A great heterogeneity is represented in intravenous fosfomycin studies, further plagued by the absence of control groups. Grabein et al were able to identify only 10 controlled studies with solely one having been published the current decade; fosfomycin had similar clinical efficacy when compared with various agents (37). Recently, the results of a multicenter, randomized, double-blind phase 2/3 trial were announced, showing non-inferiority of intravenous fosfomycin to piperacillin/tazobactam, in patients with complicated urinary tract infections (cUTIs), or acute pyelonephritis (42). These results however should be extrapolated with caution within the ICU environment, since only one in three pathogens exhibited a resistance phenotype.

Fosfomycin is not extensively used against MDR infections, with only 10 among 342 patients with bloodstream infections by CRE in the INCREMENT cohort being treated with FD (43). These data imply that FD is still regarded as salvage treatment for CR infections or treatment for breakthrough infections in patients already receiving anti-XDR treatment. Indeed, a few years ago our group was able to show that almost 2/3 of 48 critically ill patients suffering from CR infections, were already receiving colistin for a median of 13.5 days (31). Apart from the fear of resistance development during treatment, several reasons might account for FD under-prescription. Absence of high-quality evidence of efficacy could be implicated, along with difficulties of unobstructed access to FD with only few countries excepted (44). Lately FD has been approved for use in at least 14 European member states (45), while in the US ZTI-01 (Contepo^®^) has been granted Qualified Infectious Disease Product and Fast Track designations by the US Food and Drug Administration in several indications (44). In addition, several laboratory issues may stand in the way of FD use, such as the poor performance of diffusion gradient and broth microdilution techniques in comparison with gold standard agar dilution (39, 46–48), the particularity of disc diffusion susceptibility determination (49), the absence of susceptibility thresholds adapted to intravenous use (49, 50) and the difference in proposed susceptibility thresholds between CLSI and EUCAST (50, 51). Finally, labeling information in most European countries encourages salvage use (52). A few years ago our group showed that less than half of 48 fosfomycin-treated critically ill patients, received the first dose of the drug within the first 24 h from infection onset, and these patients tended to have better outcomes than their delayed initiation counterparts (31). As antimicrobial treatment exerts its maximum effect when applied early (53), surveillance cultures (54), or rapidly detection of carbapenemase production in microbiological samples (9), may early identify patients that might benefit from FD treatment.

It is undisputable that there is a place for FD within the ICU, against CR infections (8, 23). Probably, the greatest unmet need that FD is qualified to cover are XDR and PDR CRE infections, especially in the presence of colistin resistance or production of metallo-β-lactamases. Against CR pseudomonal infections, more agents are available and fosfomycin MICs are higher (44), however FD might be occasionally useful. It is questionable whether recent results implying modest *in vivo* synergistic effects with colistin against *A. baumannii* infections (28) will expand its use in this indication. Regarding specific sites of infection, it is anticipated that FD will have its best performance in the urinary tract. Nevertheless, currently available data imply comparable activity in other conditions, as well, such as ventilator-associated pneumonia (VAP) or bloodstream infections (BSIs) (31, 31). Doses up to 16–24 g/daily have been used in MDR infections, which seem justified based on pathogens MICs and fosfomycin pharmacokinetic properties (55). However, according to a recent population pharmacokinetic (PK) study, significant variance in exposure exists in critically ill patients under modern dosage regimens. This variance is not thoroughly explained by differences in renal function and identifies an area where future research is warranted (56). It is evident that an unexplored potential exists for the intravenous form of fosfomycin.

Combination treatment is the rule for CRE infections in the severely ill host, while for pseudomonal infection it is frequently recommended, even in the absence of carbapenem resistance (7, 8, 26, 57, 58). In the case of FD, combination treatment is being proposed with greater intensity, as a means to overcome selection of resistant mutants. The dogma was recently challenged by the results of the previously mentioned ZEUS study (42), showing that there might be certain patient subgroups that may inconsequently deviate from the norm of combined treatment. Regarding the companion agent choice, no specific recommendation can be made on the companion antimicrobial, since consistent synergistic patterns have not been observed (32, 59–63) and only rarely has antagonism between fosfomycin and another agent been noted (61). Recent *in vitro* evidence implies that co-administration with aminoglycosides might be beneficial in terms of efficacy, while according to older reports fosfomycin might lessen aminoglycoside toxicity (64, 65). However, the addition of nebulized amikacin-fosfomycin at a 5:2 ratio to standard treatment did not lead to improved VAP outcomes in a recent randomized-controlled trial (RCT) (66). No synergy with colistin has been demonstrated, but here is *in vitro* and *in vivo* evidence of synergy with carbapenems (61, 67, 68) and it is extremely interesting to see whether these effects are replicated (or potentiated) with modern, more active against CRE beta lactams, such as novel beta-lactam beta-lactamase inhibitor combinations.

### Tigecycline

The glycyclcycline tigecycline has been used in the last decade as a salvage treatment for infections caused by CRE and CRAB (69). Treatment outcomes have been hampered by the low serum concentrations of the drug in the approved dosing regimen and the low penetration in the epithelial lining fluid (ELF) of mechanically ventilated patients (70). A higher dose of tigecycline (HDT) (100 mg twice daily after 200 mg of loading dose) has been suggested, particularly for VAP/HAP, *A. baumannii* infections and bacteraemic infections, although all the above represent off-label use of the drug (71, 72). Decreased fibrinogen levels have been reported with augmented dose regimens of tigecycline (73). A systematic review encompassing 263 patients from 11 studies (including only one RCT), reported significantly higher rates of gastrointestinal adverse events (nausea, diarrhea and vomiting) with HDT compared to standard dose (74).

Combination treatments of tigecycline seem mandatory, given the above-mentioned characteristics of the drug and the early warnings about increased mortality, particularly in critically ill patients and when given as monotherapy (75). Before the advent of the new antimicrobials listed in the next section, possible companions were limited to colistin and meropenem (25, 69, 76). Tigecycline-colistin or -polymyxin B combinations have shown *in vitro* and *in vivo* synergy in animal model studies with KPC-2 (77–81). Real life data on colistin-tigecycline combination were captured in the INCREMENT retrospective cohort, in which high-risk patients with CRE-BSI on tigecycline-containing combinations (79 patients) had lower mortality compared with those who received colistin monotherapy (27).

Small size and heterogeneity are the rule of almost all reports of HDT in the treatment of MDR pathogens. In a study of 30 postoperative patients admitted in the ICU with two or more positive blood cultures with KPC producing *K. pneumoniae*, HDT was compared with standard dose combined with colistin in both arms. Significantly lower mortality was demonstrated in the HDT group (82). De Pascale et al. studied HDT in a cohort of patients with VAP by CRE (27 patients) or CRAB (28 patients), in which HDT was the only predictor of clinical cure (83). A retrospective study of 40 patients with BSI caused by CPKP (23 on HDT and 17 on standard-dose tigecycline combinations) found no difference in hospital mortality (84). A systematic review of 2016 compiled 25 studies (21 controlled and 5 single -arm studies) of tigecycline in the treatment of CRE infections (85). No difference in overall mortality, clinical response and microbiological response was found between tigecycline-containing arms and comparator arms. Subgroup analyses elucidated a significantly lower 30-day mortality for patients on tigecycline combination regimens than those who received monotherapy (OR = 1.83 [95% CI = 1.07–3.12; *P* = 0.03]) and other antibiotic regimens (OR = 0.59 [95% CI = 0.39–0.88; *P* = 0.01]), respectively. In addition, ICU mortality was significantly lower in HDT compared to standard dose (OR, 12.48; 95% CI, 2.06–75.43; *P* = 0.006) (85). A great heterogeneity of the studies included in this meta-analysis was reported (85, 86).

Tigecycline will continue to be a valuable antibiotic, as part of combination regimens in the new era of antibiotics (75). HDT is almost mandatory when treatment of difficult to -treat organisms is considered, probably excluding intraabdominal non-bacteremic infections. As part of empiric regimens, it can offer coverage against *A. baumannii*, depending on local epidemiological data (82). Emerging data on possible synergy with new antibiotics such as plazomicin, need further evaluation in order to define other possible combinations (87).

### Aminoglycosides

The worldwide expansion of XDR pathogens and particularly that of CRE has brought into light aminoglycosides, which may retain activity even in XDR isolates (88). *In vitro* susceptibility rates may vary significantly, depending on the dissemination of aminoglycoside modifying enzymes, which are frequently co-transferred along with other resistance genes on mobile genetic elements (89–91). It is of concern that expression of 16S rRNA methyltranferases, enzymes which confer resistance to all aminoglycosides, is especially common in NDM-producing CRE (40). In a recent multicenter study from Greece, 396 consecutive CRKP isolates were tested for susceptibility to antibiotics and mechanisms of carbapenem resistance. Gentamicin exhibited 61.9% susceptibility against this collection of strains, being higher for KPC-producers and dual KPC-VIM producers (69.1 and 90.1%, respectively); *in vitro* susceptibility against NDM-1, VIM, and OXA-48 producers was 42.6, 38.2, and 28.6%, respectively (92). Taking into account that colistin resistance was 40.6%, with the highest rates (>90%) observed among isolates exhibiting dual VIM-KPC production, it easily understood how important have become aminoglycosides in the contemporary armamentarium.

Aminoglycosides have been traditionally used as part of combination regimens and only in urinary tract infections they have been used as monotherapy. Response rates of 88% have been reported with aminoglycoside monotherapy in UTIs compared to only 64% with polymyxins (93, 94). Treatment of other body compartments is hampered by the PK/PD obstacles of the drug; monotherapies of CRE except UTIs have been associated with unacceptably high mortality rates of 80% (95). Therefore, combinations with other antibiotic(s) active against the targeted pathogen are recommended. Observational studies including large cohorts of patients with KPC-KP infections have shown that the combination of a carbapenem with an aminoglycoside was associated with the lowest mortality rate, provided that the MIC of the pathogen against meropenem was ≤8 mg/L (25, 26, 95). Aerosolised aminoglycosides may represent an important alternative for VAP, due to suboptimal PK/PD targets attained with iv administration (96, 97). Despite promising preliminary studies, RCTs failed to demonstrate improved mortality when an inhaled aminoglycoside was added as definitive treatment of HAP and VAP through an optimized device; lack of target population (i.e., MDR and XDR infections) among persons enrolled in the study may have obscured any added benefit from aerosolized antibiotics (66, 97).

Current optimal use of aminoglycosides has important drawbacks. First, established breakpoints of resistance differ between the two major relevant societies, being set at 16 mg/L (CLSI) and 8 mg/L (EUCAST) for amikacin; and 4 mg/L (CLSI) and 2 mg/L (EUCAST) for gentamicin (51, 98). Many MDR and XDR pathogens have borderline susceptibilities against these breakpoints, a fact further complicated by great the difficulty to predict PK/PDs of aminoglycosides in critically ill patients (88, 99–104). Recent reports from critically ill patients have shown that for isolates with an MIC of 16 mg/L for amikacin and 4 mg/L for gentamicin, the necessary doses to achieve therapeutic concentrations in plasma that achieve the PK/PD target would be up to 30–40 and 8–10 mg/kg for amikacin and gentamicin, respectively. Even with the more conservative EUCAST breakpoints, the PK/PD target of 8 mg/L for amikacin would not be achieved despite administration of higher doses than approved (101–103). When the desired therapeutic concentration is not in the blood but in the lung parenchyma, further PD obstacles arise (104). Given the PK/PD limitations of aminoglycosides, a high dose has been recommended followed by renal replacement therapy as a measure of minimizing associated renal toxicity (105).

Clinical experience with aminoglycosides as monotherapy or as “active monotherapy” when co-administered antibiotics were inactive *in vitro* against the targeted pathogen. However, small published series verified PK/PD considerations and consistently reported clinical failure with isolates having MICs on the breakpoint of susceptibility, whereas clinical success was demonstrated in infections by isolates with low MICs (106, 107). According to the PK/PD data listed above, monotherapy with aminoglycosides would be very risky for critically ill patients for infections in compartments outside the urinary tract (88, 93, 95, 108, 109).

Short duration courses of aminoglycosides (5–7 days) given as once-daily regimens are associated with less nephrotoxicity compared to multiple daily dosing (110, 111). Even high doses of aminoglycosides were well tolerated in terms of nephrotoxicity (101, 102). Acute kidney injury rates (AKI) between 12 and 17% have been reported in reports of critically ill patients; septic shock and prolonged administration (>10 days) were associated with increased nephrotoxicity risk (110, 112). Consequently, therapeutic drug monitoring should be undertaken, to ensure that therapeutic and non-toxic levels will be delivered to the patient. As shown in Table 2, amikacin and gentamicin are recommended to be given once daily, without a loading dose (111). Higher doses recommended for patients treated for serious CRE infections to attain a maximum serum concentration of 30 and 60 mg/ml, respectively corresponding to a PD target of 8 times the MIC of the pathogen (111, 123). Peak levels of 15–20 mg/ml for gentamicin and tobramycin and 20–40 mg/ml for amikacin are targeted with once-daily regimens. Trough levels indicative of low nephrotoxicity risk are 1 mg/ml for gentamicin or tobramycin, and 1–4 mg/ml for amikacin (111, 123).

**Table 2:** Intravenous novel and older antimicrobial agents against MDR and XDR Gram-negative pathogens (1, 8, 24–26, 59, 64, 99, 110, 113–122, 136).

**Drug**	**Dose**	**Adjustment to renal function**	**CRRT**	**Comments**
**NEW DRUGS**				
Ceftazidime/avibactam	2.5 g (2 g/0.5 g) q8h (infusion over 2 h)	CrCl >50: 2.5 g q8 h CrCl 31-50:1.25g q8h CrCl 10-30: 0.94g q12h CrCl <10: 0.94g q48h Hemodialysis: 0.94g q48h (administration after hemodialysis session)	1.25 g q8h	FDA and EMA approved for cUTI, cIAI, HAP, and VAP EMA additionally approved for aerobic Gram- negative infections in patients with limited treatment options
Ceftolozane/ Tazobactam	Dose for pneumonia (off label): 3g q8h (infusion over 1 h)	CrCl >50: 3 g q8h CrCl 30–50:1.5 g q8h CrCl 15–29: 750 mg q8h CrCl <15: no data	No data	FDA & EMA approved for cIAI (in combination with metronidazole) & cUTI, including AP Clinical trial in progress to assess the value in treatment of VAP
	Dose for other indications: 1.5g (1g/0.5g) q8h (infusion over 1h)	CrCl >50: 1.5 g q8h CrCl 30–50: 750 mg q8h CrCl 15–29: 375 mg q8h CrCl <15: 750 mg loading dose then 150 mg q8h Hemodialysis: 750 mg loading dose then 150 mg q8h (administration after hemodialysis session)	No data	
Meropenem/Vaborbactam	4g (2g/2g) q8h (infusion over 3 h)	CrCl >50: 4g q8h CrCl 30–49: 2 g q8h CrCl 15–29: 2 g q12h CrCl <15: 1 g q12h Hemodialysis: 1 g q12h (administration after hemodialysis session)	No data	FDA approved for cUTI, including AP EMA approval for cUTI, cIAI, VAP, HAP, and treatment of infections due to aerobic Gram- negatives in adults with limited treatment options
Plazomicin	15 mg/kg q24h (infusion over 30 min)	CrCl ≥60: 15 mg/kg q24h CrCl 30-60: 10 mg/kg q24h CrCl 15-29: 10mg/kg q48h CrCl <15, Hemodialysis: No data	No data	FDA approved for cUTI, including AP EMA approval pending
Eravacycline	1 mg/kg q12h (infusion over 60 min)*^a^*	No dose adjustment	No dose adjustment	FDA and EMA approved for complicated intra-abdominal infections,
**OLD DRUGS**				
Colistin	Loading dose: 9 MIU (infusion 30 min to 1 h) Maintenance dose: 4.5 MIU q12h after 12 h	Daily dose divided by two:CrCl ≥ 90: 10.9 MIU CrCl: 80 to <90: 10.3 MIU CrCl 60 to <70: 8.35 MIU CrCl 70 to <80: 9.00 MIU CrCl 50 to <60: 7.40 MIU CrCl 40 to <50: 6.65 MIU CrCl 30 to <40: 5.90 MIU CrCl 20 to <30: 5.30 MIU CrCl 10 to <20: 4.85 MIU CrCl 5 to <10: 4.40 MIU Hemodialysis: 3.95 MIU and supplementary dose 1.2–1.6 MIU for a 3- or 4-h session after the dialysis	6.5 MIU q12h	FDA approved for serious infections that are proven or strongly suspected to be caused by susceptible Gram-negative organisms EMA approval for treatment of infections caused by MDR Gram-negative pathogens with limited options Dosage proposal by International Consensus Guidelines for the Optimal Use of the Polymyxins
Polymyxin B	Loading dose: 2.5 mg/kg (1-h infusion) Maintenance dose: 1.5 mg/kg q12h (1-h infusion) after 12 h	No dose adjustment	No dose adjustment	Not available in Europe
Fosfomycin	6–8 g q8h	CrCl 40: 70% (in 2–3 divided doses) CrCl 30: 60% (in 2–3 divided doses) CrCl 20: 40% (in 2–3 divided doses) CrCl 10: 20% (in 1–2 divided doses) Hemodialysis: 2 g q48h (administration after hemodialysis session)	No dose adjustment	In combination therapy with other active drugs IV fosfomycin not FDA approved. EMA started reviewing in 2018 medicines containing fosfomycin
Gentamicin	5 mg/kg q24h (7 mg/kg q24h if critically ill)	CrCl > 80: 5 mg/kg q24h CrCl 60–80**:** 4 mg/kg q24h CrCl 40–60: 3.5 mg/kg q24h CrCl 30–40: 2.5 mg/kg q24h CrCl 20–30: 4 mg/kg q48h CrCl 10–20: 3 mg/kg q48h CrCl 0–10: 2 mg/kg q72h Hemodialysis: 2 mg/kg q72h (administration after hemodialysis session)	1.7–2 mg/kg q24h	Approved for the treatment of serious infections caused by Gram- negative and MDR infections causing cUTI. Optimal efficacy with once-daily dosing is preferable to multiple daily doses and peak levels of 8–10 mg/ml and trough levels of 1 mg/ml are desired. Aminoglycosides can be useful as part of combination regimens for treating KPC producing Enterobacteriaceae infections.
Tigecycline	Loading dose 100–200 mg, Maintenance dose: 50–100 mg q12h	No dose adjustment	No dose adjustment	FDA & EMA approval for cIAI, SSSI FDA additionally approval for CAP High-dose recommended (off-label) in critically ill patients with CRE infections and limited treatment options Inadequate serum and pulmonary drug concentrations for effective treatment of bloodstream infections and pneumonia
Meropenem	2 g q8h (extended infusion 3 h)	CrCl ≥50: 2 g q8h CrCl 30–49: 1g q8h CrCl 10–29: 1g q12h CrCl <10: 1 g q24h Hemodialysis: 1 g q24h (administration after hemodialysis session on dialysis day)	2gr q12h	In combination with another *in vitro* active drug for CRE infections (when meropenem MICs ≤8 mg/L)
Ertapenem	1 g q24h	CrCl 30–90: No dose adjustment CrCl <30: 0.5 g q24h Hemodialysis: 0.5 g q24h (administration after hemodialysis session on dialysis day)	1 gr q24h	Indicated only as part of a double-carbapenem combination for XDR and PDR Enterobacteriaceae (KPC or OXA-48 producing strains) FDA approved for cIAI, SSSI, CAP, cUTI

*AP, acute pyelonephritis; CAP, community acquired pneumonia, CrCL, creatinine clearance (calculated as ml/min/1.73m2); cIAI, complicated intra-abdominal infections; CRE, carbapenem resistant Enterobacteriaceae; SSSI, skin and skin structure infections; cUTI, complicated urinary tract infections; CRRT, continuous renal replacement therapy; EMA, European medicines agency; FDA, US food and drug administration; h, hours; HAP, hospital-acquired pneumonia; min, minutes; MDR, multidrug-resistant; MIU, million IU; PDR, pandrug resistant; q8h, every 8 hours; q12h, every 12 hours; q24h, every 24 hours; q48h, every 48 hours; VAP, ventilator-associated pneumonia; XDR, extensively drug-resistant*.

a * Increase dose to 1.5mg/kg q12h if co-administered with a strong CYP3A4 inducer: e.g. rifampicin, carbamazepine, fosphenytoin*.

When combination treatment is considered, aminoglycosides have been combined with almost all classes of antibiotics (88). Synergy of aminoglycosides with carbapenems has produced encouraging *in vitro* results in CRE isolates with resistance to carbapenems (124, 125). Further clinical evidence is important on these observations.

### Carbapenem-Containing Combinations and Double Carbapenem Combination

Before the launching of the new combinations of beta-lactams/beta-lactamase inhibitors with activity against KPC producers, carbapenems have been used as a life-saving therapeutic approach in infections caused by strains producing carbapenemases, despite the apparent paradox (25, 76). High doses and extended infusions were used (particularly for meropenem), along with other active *in vitro* agents. Studies from Greece showed that combinations including meropenem in the treatment of patients with CRKP BSIs had the lowest mortality when the MIC of the pathogen was ≤8 mg/L (19.3 vs. 35.5% in patients with carbapenem MICs > 9 mg/L) (25). Data from Italy, in a study of 36 BSIs by KPC-KP showed that meropenem-containing regimens had a significantly lower 30-days mortality rates when the isolate's meropenem MIC was 8 mg/L or less (15.8 vs. 35.2% when the MIC was at >16 mg/L) (76). Although these data derived from small observational studies, they were verified in larger cohorts. Subsequent Italian cohorts of 661 CRKP infections of various sources and 595 BSIs, respectively, highlighted the importance of high-dose meropenem combinations in achieving successful clinical outcomes with isolate's MIC against meropenem up to 16 mg/L (26, 126). Non-carbapenem regimens were associated with worse outcomes across all these studies, particularly when they consisted of monotherapies (25, 76, 127).

Interestingly, multidrug carbapenem-containing combinations were most effective in patients at high risk for mortality (i.e., those in septic shock (25). Based on the experience gathered from KPC-KP infections in Italy and Greece, in a position document, 13 experts from these two countries concluded that “administration of high-dose (e.g., 2 g every hours), prolonged infusion meropenem could be beneficial in KPC-KP infections if MIC is ≤8 mg/L,” whereas for MICs up to 32–64 mg/L, “meropenem administration should be considered if therapeutic drug monitoring is available to monitor optimal drug exposure” (8). With the advent of the novel antibiotics with activity against KPC-KP, carbapenems are no longer preferable to a beta lactam antibiotic against which the isolate is susceptible. However, early studies have demonstrated similar effects of carbapenem-containing regimens on mortality in severe infections caused by VIM-1 producing *K. pneumoniae*, provided that the carbapenem MIC of the isolate was <4 mg/L (127). Since we don't have new antibiotics active against MBL-producers, experience acquired so far could be still life-saving against XDR isolates, particularly when the underlying mechanism of resistance is VIM or the isolate coproduces KPC and VIM. Further evaluation is needed and rapid communication of carbapenem MICs to the treating physicians, irrespective of the mechanism of resistance produced by the recovered pathogen.

Double carbapenem combination (DCC) has emerged as a salvage treatment option against XDR and PDR CRE, based on a preliminary concept study by Bulik and Nicolau on the combination of ertapenem and doripenem (128). They're *in vitro* and *in vivo* observations, suggested that ertapenem acts as a “suicide inhibitor of KPCs” due to its higher affinity to the enzyme, leaving the other carbapenem intact and active against the isolate (128). Further *in vitro* studies showed that only KPC and not MBL enzymes respond to this maneuver and various carbapenem combinations were proven successful *in vitro* (129). These observations were verified in small observational studies with inherent biases (lack of randomization and controls) (23, 130–132). A recent publication summarized the experience among 211 patients treated with this modality in observational studies (133). The most common combination was ertapenem (1 g q24h IV, infused over 30 min) plus meropenem (2 g q8 h, 3 h infusion). The administration of other antibiotics in almost one third of cases (mostly colistin) obscures the evaluation of DCC *per se*, however, a clinical success rate of 70% with all-cause mortality of 26% seems promising in a population of critically ill patients (50% bacteraemic). It is noteworthy that in a study encompassing 27 patients, the majority of whom received only DCC, PDR infections had a successful clinical and microbiological outcome in 78.5% (131). In a similar study in which the authors preferred triple drug combination with colistin, similar clinical efficacy was shown (75%) (132). In the single study employing a case control approach, 48 patients receiving DCC were compared to 96 control patients who were administered standard treatment (i.e., colistin, tigecycline, aminoglycoside) for documented infections by CPKP. DCC was associated with significantly lower 28-days mortality (47.9 vs. 29.2%, *p* = 0.04) and was shown to have a protective effect on mortality in the multivariate analysis (OR: 0.33, 0.13–0.87) (133).

Administration of a double carbapenem (with or without colistin) seems a promising salvage treatment in infections caused by PDR isolates harboring serine-carbapenemases. *In vitro* data showed synergistic activity of DCC against OXA-48 producing MDR and XDR *K. pneumoniae* (134). Finally, two kidney transplant recipients were reportedly cured with DCC plus oral fosfomycin from urinary tract infections caused by NDM-harboring Enterobacteriaceae (135). Further randomized controlled trials are needed to assess DCC as salvage treatment in infections by XDR pathogens exhibiting various mechanisms of resistance to carbapenems.

## New Antibiotics

### Ceftolozane-Tazobactam

Ceftolozane/tazobactam (C/T) is a combination of a β-lactamase inhibitor in use for decades, with a new oxyimino-cephalosporin with structural similarity to ceftazidime. It possesses a potent activity against *P. aeruginosa*, due to the inherent high affinity of ceftolozane for the penicillin-binding proteins that are essential for these species (113, 136). Common mechanisms of resistance in *P. aeruginosa* (Table 1) such as overexpression of efflux pumps, loss of the outer membrane porin OprD and AmpC overproduction do not affect ceftolozane, whereas development of resistance to C/T requires several mutations leading to an overexpression of AmpC enzymes and structural modifications (113). Tazobactam offers stability against most ESBLs (136). C/T is being hydrolyzed by carbapenemases (KPC, VIM, NDM, GES) with possible exception OXA-48 against which the combination may retain some activity (113). *In vitro* studies have shown an excellent *in vitro* activity against *P. aeruginosa* and Enterobacteriaceae. Of interest for ICU patients is also a demonstrated *in vitro* susceptibility against *Burkholderia* spp. and *Stenotrophomonas maltophilia* isolates with relatively low MICs, but no substantial susceptibility against *A. baumannii* (113, 137, 138). C/T has considerable activity against *Streptococcus* spp (except for *S. pneumoniae*), no activity against staphylococci and enterococci and minimal activity against anaerobes (113, 137–139). Time kill studies have verified that C/T is the most potent antibiotic against susceptible *P. aeruginosa*, whereas carbapenems were more cidal against Enterobacteriaceae (140). Recent data from 53 ICU VAP infections by *P. aeruginosa* showed that C/T exhibited the most potent *in vitro* activity compared to ceftazidime/avibactam, imipenem and ciprofloxacin (141).

C/T is approved in the US and Europe for cIAIs and cUTIs based on two phase 3 registration trials which showed non-inferiority of C/T (with metronidazole) vs. meropenem in cIAIs and vs. levofloxacin in cUTIs (142, 143). Results from a phase 3 clinical trial in HAP requiring mechanical ventilation and VAP (ASPECT-NP) are anticipated to support the approval of the relevant indication. Double C/T dosage (3 g every 8 h for normal renal function) was employed in the trial of nosocomial pneumonia, based on PK/PD studies showing that with this dosage there is more than 90% probability of PD target attainment for pathogens with MIC ≤8 mg/L, well below the breakpoint of susceptibility for *P. aeruginosa* which was set by both CLSI and EUCAST at 4 mg/L (144, 145). Dosage in impaired renal function is displayed in Table 2 (146–149). Approved dosage corresponds to target attainment for pathogens with an MIC of 4 mg/L in patients with normal renal function or augmented renal clearance (146) and 8 mg/L in patients with impaired renal clearance. Despite this, data from the phase 3 trial investigating cIAIs showed that patients with moderate renal impairment (CrCl 30–50 mL/min) had lower cure rates in the C/T plus metronidazole arm compared to the meropenem arm (48 vs. 69.2%, respectively) (142). Following these reports daily monitoring of renal function in patients with unstable renal function and re-adjustment of C/T dosage according to creatinine clearance measurement is strongly recommended. Although the labeled duration of the infusion is 1 h, a 4–5-hours extended infusion may provide better probability of PD target attainment for pathogens with elevated MICs (147, 150).

Currently available data on the clinical effectiveness of C/T for respiratory infections and VAP derive from case series and case-reports with variable dosage regimens and in combination with other antibiotics. An overall clinical success of 61.4% among patients with *P. aeruginosa* pneumonia treated with C/T is estimated (151–156), whereas a clinical efficacy of 71% was reported a single study of infections by MDR *P. aeruginosa* (152). Clinical failures have been associated with an MIC higher than 4 mg/L and the use of the lower dosage regimen (157). Of concern are reports of resistance to C/T during therapy of *P. aeruginosa* infections (152, 155, 158). A recent retrospective report on 101 infections by *P. aeruginosa* (50% of which XDR), showed for ceftolozane/tazobactam (35% in combination) an overall clinical success of 83.2%. Continuous renal replacement therapy and sepsis were identified as factors associated with lower success rates (159).

The clinical effectiveness of C/T against ESBL-producing Enterobacteriaceae, was estimated in a secondary analysis of the clinical trials of cIAIs and cUTIs; clinical cure rates of 97.4% were reported for C/T in *E. coli* or *K. pneumoniae* infections, compared with 82.6 and 88.5% for levofloxacin and meropenem, respectively (114).

C/T is a valuable addition to our armamentarium, as the most potent antibiotic against *P. aeruginosa* infections with additional activity against ESBL-producers. The main place in therapy of C/T would be the empirical or definitive treatment of infections suspected or caused by *P. aeruginosa* (**Figure 3**), in which it could serve as a beta-lactam backbone of treatment. In settings where carbapenemase production is low among *P. aeruginosa* strains, C/T can provide a reliable empirical coverage (160). In patients with various risk factors for MDR (hematologic malignancy, prolonged hospitalization, prior ICU admission, previous receipt of other anti-pseudomonal agents etc.), C/T can be used as part of an empirical antibiotic regimen, along with a second agent, to ensure adequate coverage (136, 161). Pending the results of the phase 3 study of nosocomial pneumonia, C/T will probably hold an important role in the reiteration of guidelines for VAP caused by *P. aeruginosa* (162). In infections by CR-*P. aeruginosa* without carbapenemase production, C/T should be the drug of choice. C/T holds also an important role against ESBL-producing Enterobacteriaceae, where it represents a reliable a carbapenem-sparing option.

### Ceftazidime-Avibactam

Avibactam, a novel non-β-lactam, β-lactamase inhibitor, restores the activity of ceftazidime against the majority of β- lactamases (ESBLs and carbapenemases, including KPCs– Ambler Class A, AmpC —Class C and oxacillinase OXA-48—Class D), resulting in extended spectrum of CAZ-AVI combination against a wide range of MDR bacteria (115, 163). Notably, avibactam is not able to inhibit strains producing metallo-β-lactamases (MBL—Class B), as well as many of the Class D enzymes (115). CAZ-AVI is FDA and EMA approved for the treatment of complicated intra-abdominal infections (cIAIs), complicated urinary tract infections (cUTIs), hospital-acquired, and ventilator associated pneumonia (HAP/VAP) and (EMA only) infections due to aerobic Gram-negative organisms in adult patients with limited treatment options (116, 164).

The International Network For Optimal Resistance Monitoring (INFORM) global surveillance program from 2012 to 2015 demonstrated 99.4 and 98.5% susceptibility to CAZ-AVI for all Enterobacteriaceae isolates and for meropenem-non-susceptible, MBL-negative isolates, respectively (16, 17). In a recent surveillance study of 394 clinical isolates of CR-KP from Greece, CAZ-AVI inhibited 99.6% of KPC and 100% of OXA-48-like-producing isolates (40); data from US were similar, showing 98.7% *in vitro* susceptibility of CAZ-AVI against KPC-producing *K. pneumoniae* isolates (165). Although only few studies have differentiated activity of CAZ-AVI against KPC subtypes, emerging data indicate that KPC-3-producing strains have higher MICs than KPC-2 producers. In a recent study KPC-3 strains exhibited a ceftazidime-avibactam MIC of 8 μg/ml; furthermore KPC-3 possessed 30-fold greater hydrolytic activity against ceftazidime than KPC-2 (166, 167). According to European Committee on Antimicrobial Susceptibility Testing (EUCAST) (51) and Clinical Laboratory Standards Institute (CLSI) (98). Minimum Inhibitory Concentration (MIC) breakpoints for CAZ-AVI is ≤8 mg/L for both Enterobacteriaceae and *P. aeruginosa* species.

KPC-producing strains may harbor additional resistance mechanisms, including overexpression of efflux-pump, mutations in porin genes, production of multiple carbapenemases and *de novo* mutations in the blaKPC-3 gene (168–170). Emerging resistance to CAZ-AVI has been attributed to the expression of KPC variants with substitutions in the omega-loop; this knowledge has fueled the research for a new active formulation of β-lactamase inhibitors, that will potentially overcome this new resistance mechanism (171, 172). These KPC variants seem to lose their carbapenemase activity and exhibit a meropenem susceptible phenotype (173). Unfortunately, restored meropenem susceptibility is not permanent, since the isolate after exposure to meropenem rapidly returns to the initial KPC-expressing status along with the emergence of porin mutations (174). In some early reports, resistance of *K. pneumoniae* to CAZ-AVI (MIC ≥16 mg/L) was observed with short courses of therapy, especially after monotherapy, reflecting the need for vigilance in clinical practice (168). As a result of these reports, some experts suggest combination treatments in order to prevent emergence of resistance (8).

*In vitro* activity of CAZ-AVI against *P. aeruginosa* varies across studies, owing to the different underlying mechanisms of resistance, compared to Enterobacteriaceae (175). Data from recent studies showed higher *in vitro* activity of CAZ-AVI compared to ceftazidime, piperacillin-tazobactam or meropenem when tested against both MDR and XDR, *P. aeruginosa* strains (175–177). A susceptibility rate as high as 85.1% for meropenem-non-susceptible *P. aeruginosa* has been reported, rendering CAZ-AVI the second most potent *in vitro* agent after colistin; however, in other studies *in vitro* activity around 50% was shown for isolates resistant to ceftazidime and meropenem (16, 17, 176, 177). Differences in susceptibility among strains of *P. aeruginosa* to β-lactam agents, including CAZ-AVI, may be attributed to a variable prevalence of the metallo-β-lactamases, such as VIM-2 (176–178). CAZ-AVI combination is only slightly active against *A. baumannii* (due to avibactam's vulnerability to OXA-type carbapenemases frequently carried by *A. baumannii* species). Marginal activity against anaerobic Gram-negative bacteria mandates addition of metronidazole in intraabdominal infections; finally, CAZ-AVI is inactive against Gram-positive cocci (173, 176).

PK/PD studies for both ceftazidime and avibactam revealed linear pharmacokinetics, glomerular excretion and human protein binding 10 and 8%, respectively (116, 164, 179, 180). As demonstrated by PK/PD studies in humans, ceftazidime and avibactam equally penetrate into human bronchial epithelial lining fluid (ELF), with AUC values in ELF ~30% of those in plasma (181). The recommended dosage of CAZ-AVI is 2/0.5 g every 8 h by intravenous infusion over 120 min. Dose adjustment to impaired renal function is shown in Table 2 (116, 164).

Two identical, randomized Phase III trials (RECLAIM I and II) compared CAZ-AVI plus metronidazole with meropenem in patients with cIAIs, and proved non-inferiority (182). In the subgroup of patients with impaired renal function, clinical cure rates were higher with meropenem, arguing for caution with dosage regimens in patients with unstable renal function. Subgroup analysis of patients with ceftazidime-resistant bacterial infections, showing clinical cure rates similar to meropenem (83.0 vs. 85.9%, respectively) (16, 17). In line were the outcomes reported from the RECLAIM III, which enrolled 432 patients from Asia (183). The REPROVE study, enrolling 879 patients with nosocomial pneumonia, including VAP, proved non-inferiority of CAZ-AVI compared to meropenem; again, efficacy of CAZ-AVI against ceftazidime-non-susceptible pathogens was comparable to meropenem (184). Two Phase III trials (RECAPTURE I, II), proved non-inferiority of CAZ-AVI to doripenem in patients with cUTIs (185). REPRISE study, a prospective, pathogen-directed (ceftazidime-resistant, Enterobacteriaceae or *P. aeruginosa*), open-label, phase 3 trial, demonstrated the efficacy and safety of treatment with CAZ-AVI vs. best available therapy in cUTIs or cIAIs (186), establishing CAZ-AVI as a potential alternative to carbapenems in ceftazidime-resistant strains.

An excellent safety and tolerability profile has been shown for CAZ-AVI, with mild main adverse events, including hypersensitivity reactions, headache, nausea, constipation, and diarrhea (164, 183, 187). Physicians should take into consideration the possibility of positive Coombs direct test without documented haemolysis (3.2–20.8%) (182–186).

Post market studies showed that CAZ-AVI was mostly administered as treatment for CRE infections, with clinical response rates between 55–85%, relapse rates of 23% and a mortality rate of 10–39.5% (170, 187–194). Real life data render CAZ-AVI a “game changer” in the treatment of KPC-producers. Compared to colistin, in a prospective study of 38 patients treated with CAZ-AVI vs. 99 treated with colistin, the first group had a 64% probability of a better outcome at 30 days of treatment (193). Another single-center observational study in KPC-bacteraemic patients receiving CAZ-AVI vs. other regimens, showed clinical success more frequently with CAZ-AVI in terms of higher corresponding rates and mortality (190). In addition, a promising role for CAZ-AVI was elucidated from a retrospective analysis of the compassionate use program of CAZ-AVI from Italy; among patients with CRKP bacteraemic infections those treated with CAZ-AVI had significantly lower mortality compared with patients receiving other regimes (192). Furthermore, in multivariable analysis of 208 patients with CRKP BSI, receipt of CAZ-AVI was the only independent predictor of survival, whereas septic shock, neutropenia, Charlson score index >3. and recent mechanical ventilation were independent predictors of mortality (192). Patient's severity of disease, as displayed by the INCREMENT CPE score > 7 (193) was demonstrated as risk factor of mortality in another study, whereas pneumonia and renal replacement therapy have been elucidated as factors associated with treatment failure of CAZ-AVI treatment (190). Finally, the results of a recently published meta-analysis encompassing 12 studies and 4,951 patients, further support that CAZ-AVI is as effective as carbapenems with equivalent safety. In particular, significantly increased cure rates were achieved with CAZ-AVI in infections from resistant causative microorganisms (RR = 1.61; 95% CI, 1.13–2.29); reduced mortality was also reported (RR = 0.29; 95% CI, 0.13–0.63). Similar results were reported for cUTIs and BSI (195).

**Figure 1:**
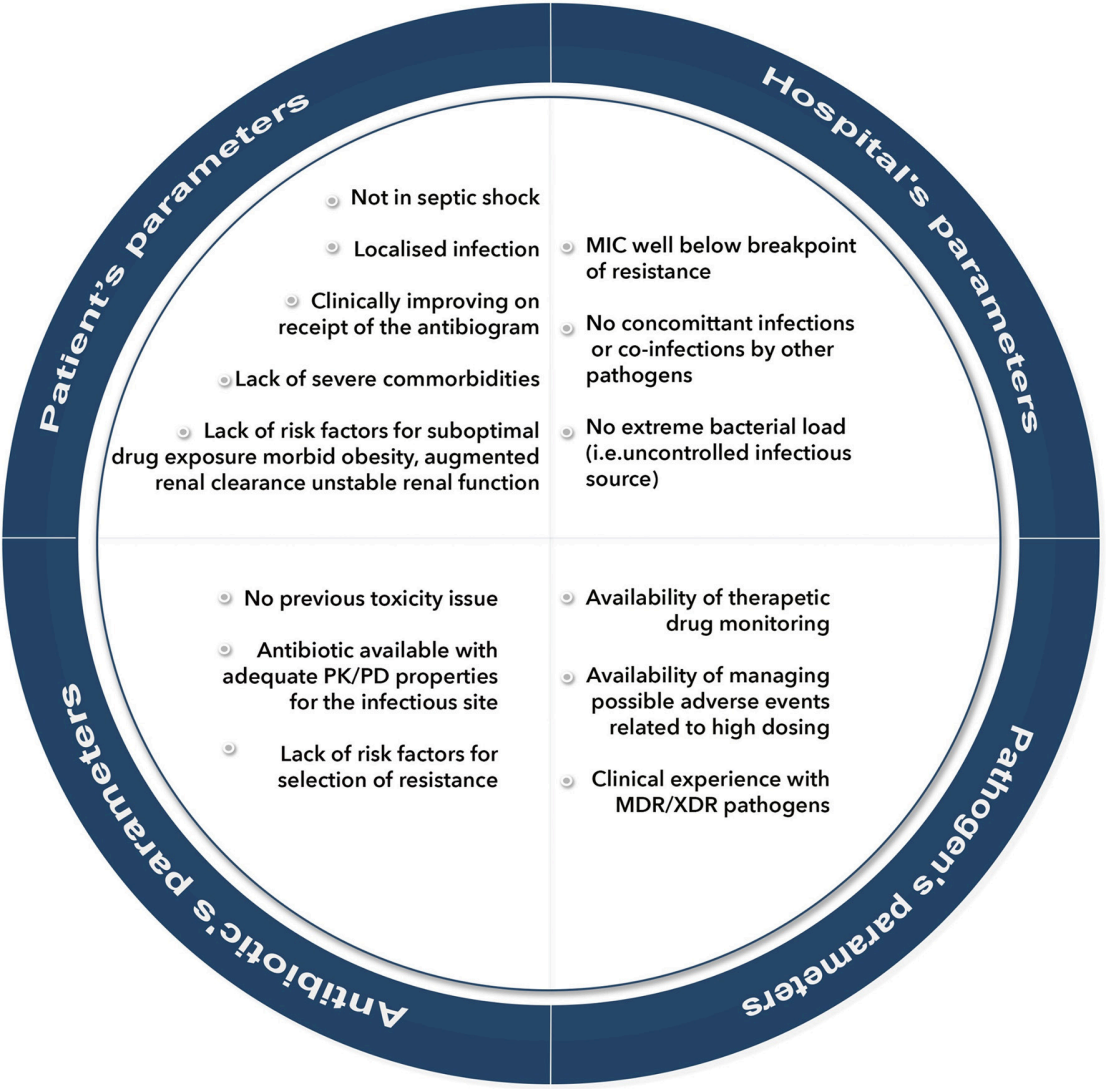
Prerequisite conditions for selecting monotherapy as definitive treatment of infections by extensively-drug-resistant (XDR) pathogens. PK/PD, pharmacokinetic/pharmacodynamic; MDR, multi-drug -resistant; MIC, minimum inhibitory concentration; XDR, extensively-drug-resistant.

**Figure 2:**
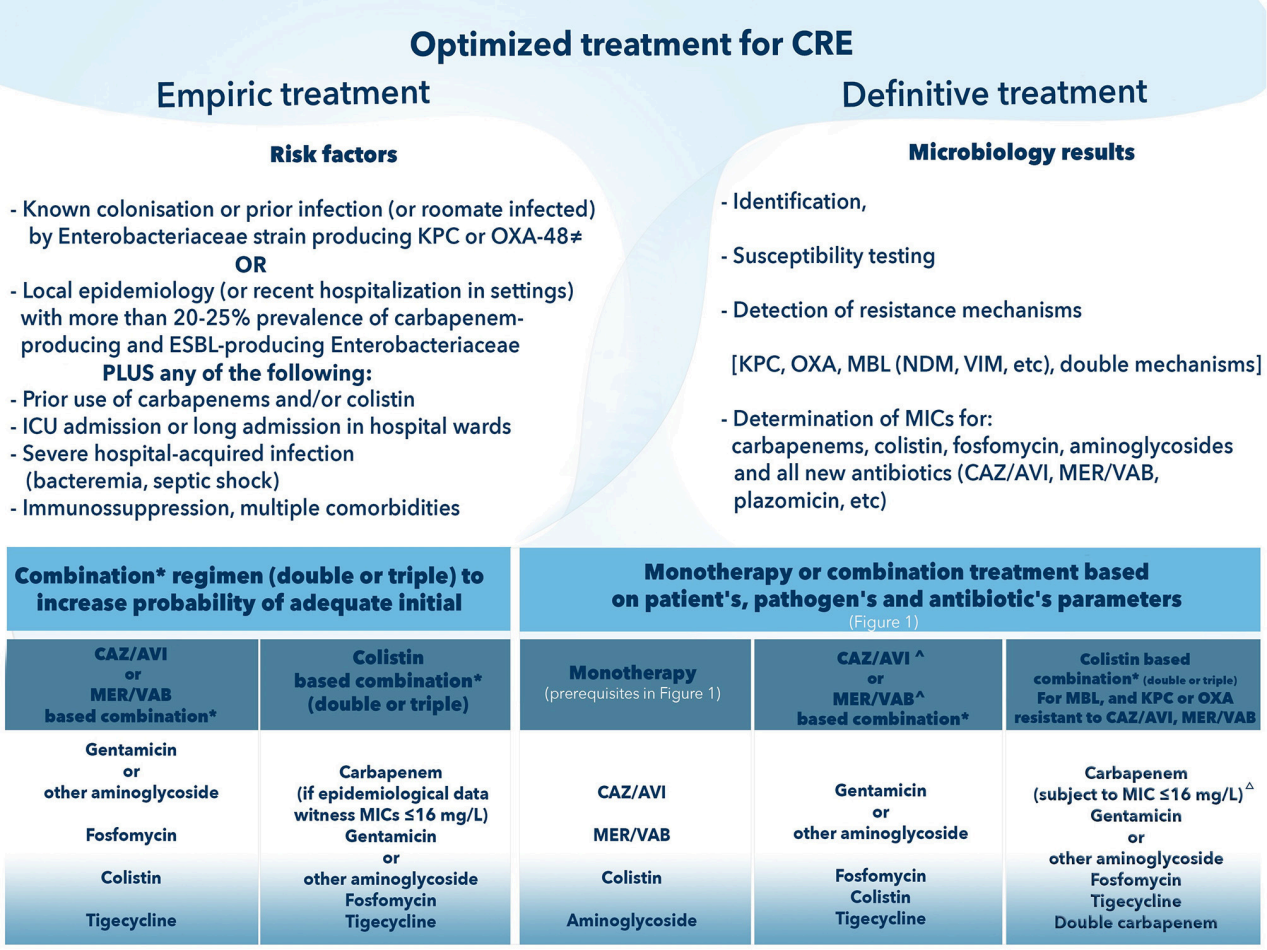
How to optimize treatment of Carbapenem-Resistant Enterobacteriaceae (CRE). CAZ-AVI, ceftazidime avibactam; CRE, carbapenem-resistant Enterobacteriaceae; MER/VAB, meropenem vaborbactam; MIC, minimum inhibitory concentration. ≠ OXA-48 is permissive only for CAZ-AVI. ^*^Components of the combination will be based on: (i) epidemiology data (for empirical regimen); (ii) pharmacokinetic/pharmacodynamic considerations relating to the source of infection; (iii) lower MIC (if possible, avoidance of antibiotics with borderline susceptibility). ∧ Selection of CAZ-AVI or MER/VAB in definitive treatment precludes demonstrated *in vitro* susceptibility and absence of detected metallo-beta lactamase mechanism of resistance; for MER/VAB absence of OXA as well. ^**Δ**^ Higher MICs against meropenem (up to 64 mg/L) may require higher doses and therapeutic drug monitoring.

Summarizing, CAZ-AVI stands out as one of the most important additions in our armamentarium, as the first marketed fixed combination with activity against KPC and OXA producers. Post market reports, reflecting real-life use are very encouraging, in terms of safety, clinical response, and survival. Currently, we do not have enough evidence to relay on monotherapy with CAZ-AVI, since previous reports on CRE infections from observational studies consistently favored combination treatments (26, 57). Expert-driven recommendations issued by relevant Italian and Greek ID Societies, support combination treatment (with an aminoglycoside including plazomicin, or fosfomycin, tigecycline, colistin). However, in non- life-threatening infections, monotherapy could be considered as part of a definitive treatment (8). The prerequisite conditions for monotherapy are illustrated in Figure 1. A proposed algorithm for the optimization of treatments of CRE is shown in Figure 2. Companion drugs of CAZ-AVI can be selected based on the antibiogram and the required PK/PD parameters in the infectious focus (8). The broad spectrum covering also ESBL-producing Enterobacteriaceae and significant proportions of *P. aeruginosa*, makes CAZ-AVI a strong component of empiric regimens in patients with risk factors for MDR infections. When designing an adequate empiric regimen, the probability of MBL-producing Enterobacteriaceae or *A. baumannii* should be balanced according to local epidemiological data and covered with a second antibiotic (an aminoglycoside, colistin, tigecycline, or fosfomycin). We believe that empiric use of CAZ-AVI should be reserved for patients with strong risk factors for infections by KPC- or OXA-48- producers and audited by antibiotic stewardship teams to avoid irrational use of this antibiotic combination (Figure 2).

### Meropenem-vaborbactam

Varobactam, previously referred as RPX7009, is a boronic acid, non-β-lactam β-lactamase inhibitor with no antibacterial activity. The addition of varobactam to meropenem, restored the activity of meropenem against KPC-producing strains due to its high affinity for serine proteases. Nevertheless, vaborbactam possesses minimal or no activity against carbapenemases of Amber class B (metallo-β-lactamases/MBLs) (196–198). More specifically, vaborbactam inhibits various class A carbapenemases (KPC-2, KPC-3, KPC-4, BKC-1, FRI-1, and SME-2), class A ESBLs (CTXM, SHV, and TEM), and class C cephalosporinases (CMY, P99), while its activity against class D carbapenemases (OXA- 48-like) is minimal (199, 200). The combination provided no additional *in vitro* activity against *P. aeruginosa, Acinetobacter* spp. and *Stenotrophomonas maltophilia* compared to meropenem alone (199).

Meropenem/vaborbactam became the first available carbapenem/ β-lactamase inhibitor for clinical use. It has shown an excellent tolerability profile and no safety concerns, with main adverse events headache, diarrhea and catheter site complications, while mild lethargy was observed in the highest-dose group (201, 202). No seizures were reported from clinical trials, however, concomitant administration of valproic acid, and other drugs medications that may compete with meropenem for active tubular secretion, such as probenecid, is not advisable (117).

In a study that evaluated the activity of meropenem/varobactam against Gram-negative strains, including MDR and XDR Enterobacteriaceae, meropenem-vaborbactam at concentration ≤2 mg/L inhibited 133 of the 135 KPC-producing isolates, while all isolates were inhibited at ≤8 mg/L (199).

In a similar study, meropenem/vaborbactam inhibited 99.0% of KPC-positive isolates of Enterobacteriaceae at ≤4 mg/L and compared to CAZ-AVI and tigecycline had equivalent—if no superior—*in vitro* activity (meropenem-vaborbactam at MIC90: 1 mg/L, was four times more potent than CAZ-AVI and at least 64-fold greater than meropenem alone) (203). In another *in vitro* study of CRE, MICs of meropenem/vaborbactam were elevated for isolates producing MBLs (MIC, 16->64 mg/L); isolates with decreased expression of porin OmpK37 or hyperexpression of the AcrAB-TolC efflux system had also increased MICs(16 mg/L) (198). Interestingly, *in vitro* studies showed similar MIC distributions of meropenem/vaborbactam for isolates producing KPC-2 and KPC-3 (203).

Meropenem/varobactam obtained FDA approval for cUTIs including pyelonephritis (117), after completion of a phase 3 study (TANGO I) demonstrating non-inferiority compared to piperacillin-tazobactam in cUTIs, including pyelonephritis (204). The currently tested dosage, approved for clinical use is 2gr−2gr every 8 h and is mainly excreted in urine (202). Another multicentre phase 3 rial (TANGO II), compared meropenem/varobactam to best available treatment (mono/combination therapy with polymyxins, carbapenems, aminoglycosides, tigecycline; or ceftazidime-avibactam alone) in infections caused by CRE—including HAP, VAP, cIAIs, cUTIs, and bacteraemia (205). The study enrolled 77 patients, 47 of whom fulfilled criteria for primary analysis. The study showed increased clinical cure rates 65.6 (21/32) and 33.3% (5/15) [95% confidence interval (CI) of difference, 3.3% to 61.3%; *P* = 0.03)] at End of Treatment and decreased mortality 15.6 (5/32) and 33.3% (5/15) (95% CI of difference,−44.7 to 9.3%). Equally important is the reduced nephrotoxicity in the arm of meropenem/varbobactam. Exploratory risk-benefit analyses of composite clinical failure or nephrotoxicity favored meropenem-vaborbactam vs. best available treatment (31.3 [10/32] vs. 80.0% [12/15]; 95% CI of difference, −74.6 to −22.9%; *P* < 0.001) (205). Based on these additional studies, in November 2018 EMA approved meropenem-varbobactam for use in “adult patients with CIAI, cUTI, HAP, VAP, bacteraemia that occurs in association with any of these infections, and infections due to aerobic gram-negative organisms where treatment options are limited” (206). TANGO III trial assessing meropenem-vaborbactam vs. piperacillin/tazobactam in patients with HAP and VAP, estimated to finish in 2020 was withdrawn (207).

Scarce published data exists on real-life experience with meropenem/vaborbactam, which is extremely important to define its real position in clinical practice. Among its major advantages is the potent *in vitro* activity against KPC-producers and the low potential for resistance development. It represents an important player in the battle against CRE mediated by KPC production.

### Plazomicin

Plazomicin is a next-generation semisynthetic aminoglycoside. As all aminoglycosides, it acts by inhibiting bacterial protein synthesis. Plazomicin is active against MDR Enterobacteriaceae, due to its stability against strains that express aminoglycoside-modifying enzymes (208). However, it is vulnerable to ribosomal ribonucleic acid (rRNA) methyltransferase enzymes which were already identified in Enterobacteriaceae, *P. aeruginosa*, and *A. baumannii*, particularly among Enterobacteriaceae harboring NDM-1 carbapenemases. These enzymes confer broad-spectrum resistance to all aminoglycosides, including plazomicin; their identification before plazomicin's introduction into clinical practice is of concern (209–212). A major advantage of plazomicin is its dose-dependent bactericidal activity. Its antibacterial spectrum includes Gram-negative bacteria, such as a wide range of Enterobacteriaceae (including CRE, ESBL, and MDR isolates) irrespective of resistance to currently available aminoglycosides (209, 211, 213). Collections of clinically important KPC-producers with resistance to aminoglycosides were inhibited by plazomicin displaying an MIC90 of ≤2 or 4 mg/L (210, 213). In a recent *in vitro* study of 300 CRE *K. pneumoniae* isolates, susceptibility to plazomicin was 87.0% (≤2 mg/L) with MIC50/MIC90 of 0.5/4 mg/L; rRNA methyltransferases (mostly rmtB) were found in an alarming 8% of isolates, never exposed to plazomicin (40).

Although plazomicin is less potent *in vitro* against non-fermenters compared to Enterobacteriaceae, OXA-producing *A. baumannii* resistant to other aminoglycosides may be susceptible to plazomicin (211, 212). Tested against 82 isolates of CRE, plazomicin showed 79% *in vitro* susceptibility with MICs ≤2 mg/L, including isolates producing metallo-β-lactamases type VIM or IMP but not NDM-1, due to the co-production of ribosomal methyltransferases (209). Anti-MRSA activity offers an extra advantage when plazomicin is part of empirical regimens in severely ill patients in settings with MRSA prevalence, despite resistance to previous generation aminoglycosides (211). Plazomicin demonstrated *in vitro* activity with MIC90 of ≤4mg/L against isolates of polymyxin-resistant Enterobacteriaceae, including mcr-1 producing isolates (214).

Plazomicin displays a linear and dose-proportional pharmacokinetic profile, with an elimination half time (t1/2) of 4 h and lack of accumulation indicative of once daily therapy. Therapeutic dosage of plazomicin was set at 15 mg/kg (118, 215).

EPIC (Evaluating Plazomicin in cUTI), was the first phase 3 registration trial (NCT02486627), in which plazomicin compared to meropenem in cUTIs met the non-inferiority endpoint (216). The second phase 3, CARE descriptive trial (ClinicalTrials.gov Identifier NCT01970371), published in January 2019, compared plazomicin vs. colistin as part of a definitive combination regimen in serious infections due to CRE excluding NDM-producers (217). CARE enrolled 39 patients with a variety of infections including 29 bloodstream infections (BSI), and 8 HAP/VAP; the study was prematurely terminated due to low enrolment. Patients received plazomicin 15 mg/kg every 24 h or colistin, in both arms combined with a second agent (tigecycline or meropenem). Using a composite endpoint, patients who received plazomicin had fewer primary end-point events [4 of 17 patients (24%) compared to 10 of 20 patients (50%) who received colistin (difference, −26 percentage points; 95% confidence interval [CI], −55 to 6). The same applied for bloodstream infections but not for VAP/HAP, probably due to the low number of patients with the latter infection. Less deaths were observed at day 14 among patients who received plazomicin-based treatment, and this trend continued through day 60. The group of plazomicin had fewer adverse events and fewer patients with clinically significant increase in serum creatinine at any point of the trial, demonstrating a safer profile against the comparator colistin (217–219).

Plazomicin was granted FDA approval in June 2018 for patients 18 years of age or older with the indication of cUTIs, including pyelonephritis caused by the following susceptible microorganism(s): *Escherichia coli, Klebsiella pneumoniae, Proteus mirabilis*, and *Enterobacter cloacae*. In the drug's SPC it is advised to be reserved as salvage approach for cUTI patients who have limited or no alternative treatment options. Potential nephrotoxicity, ototoxicity, neuromuscular blockade, and fetal harm have been included in a boxed warning despite scarce data from clinical trials (119, 219). EMA approval is pending.

**Figure 3:**
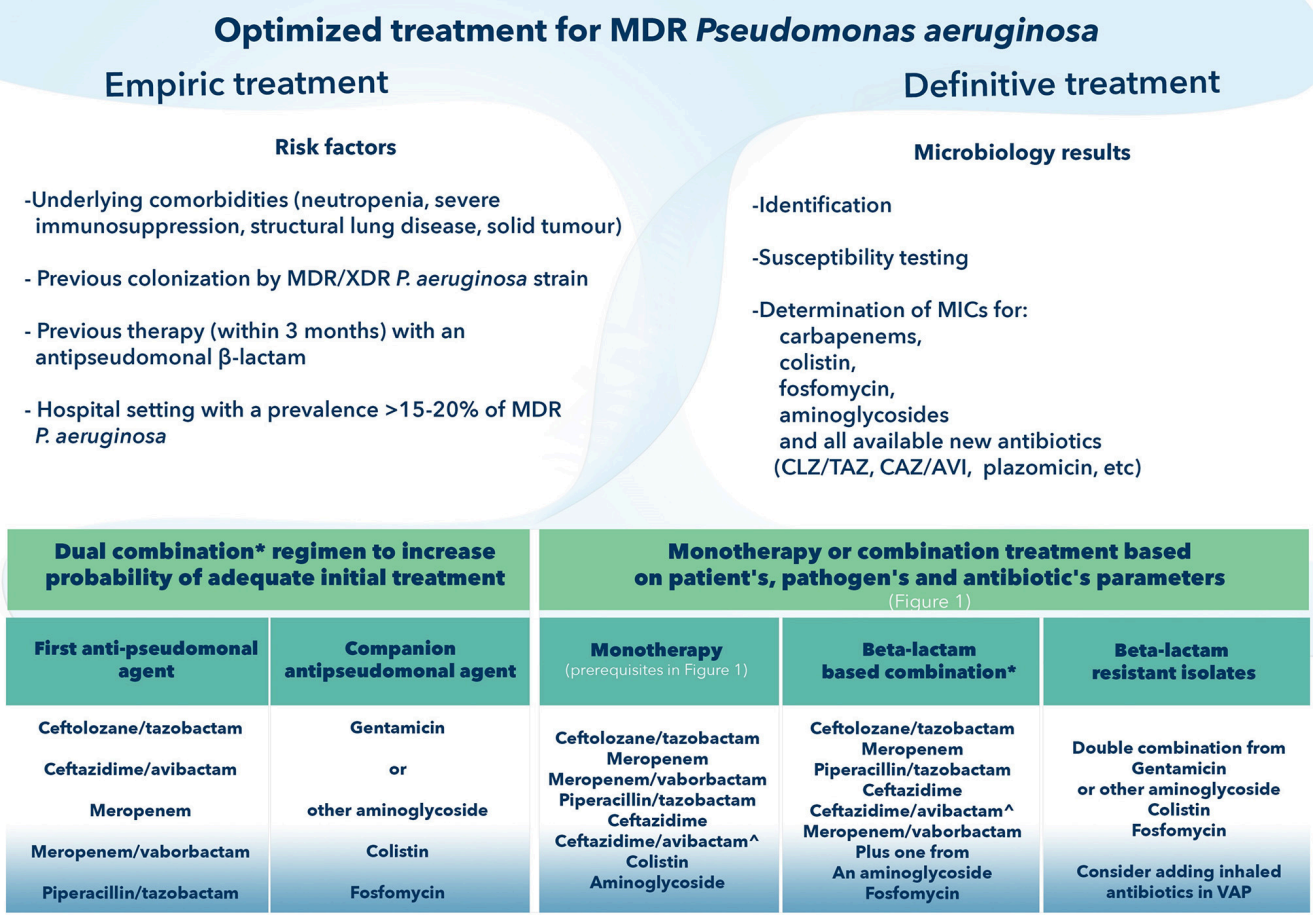
How to optimize treatment of Multi-drug-resistant *Pseudomonas aeruginosa*. CAZ-AVI, ceftazidime avibactam; CLZ/TAZ, ceftolozane tazobactam; MDR, Multi-drug resistant; MIC, minimum inhibitory concentration; XDR, extensively drug-resistant; VAP, ventilator-associated pneumonia. ^*^Components of the combination will be based on: (i) epidemiology data (for empirical regimen); (ii) pharmacokinetic/pharmacodynamic considerations relating to the source of infection; (iii) lower MIC (if possible, avoidance of antibiotics with borderline susceptibility). ∧ Selection of CAZ-AVI in definitive treatment precludes demonstrated *in vitro* susceptibility and absence of detected metallo-beta lactamase mechanism of resistance.

In summary, plazomicin a novel aminoglycoside with minor toxicity issues and a challenging antimicrobial spectrum holds promise in the battle against difficult to treat organisms. Its favorable lung penetration make it a candidate for treatment regimens of VAP, particularly as part of empiric regimens or as definitive combination treatment where monotherapy is not advised. Conceptually, aminoglycosides are not used as monotherapy; in that sense, plazomicin could be a perfect companion to the new beta-lactam-beta-lactamase inhibitors. As part of an initial empiric regimen, depending on local epidemiological data it could replace colistin in pulmonary infections due to the poor pharmacokinetics of the latter in the lung, given as companion to a tailored beta-lactam backbone (Figures 2, 3). More data on combinations and particularly on monotherapy need to be compiled. Aminoglycosides hold a pivotal role in the treatment of UTIs, with the potential of monotherapy; plazomicin, with demonstrated low rates of renal toxicity could be an option for a targeted monotherapy in XDR pathogens causing UTIs. Such an approach would spare other advanced antimicrobials.

### Eravacycline

Eravacycline is a synthetic fluorocycline, with similarities to tigecycline in mechanism of action, structure and antibacterial spectrum, including: Gram-negative bacilli (*P. aeruginosa*- excluded), regardless of resistance to other antibiotic classes (ESBL and KPC- producing Enterobacteriaceae, MDR *A. baumannii*) and Gram-positives (staphylococci, including methicillin-resistant *Staphylococcus aureus*/MRSA; enterococci, including Vancomycin-resistant *Enterococcus*/VRE) (220, 221). Potential advantages over tigecycline feature a more potent *in vitro* activity for both Gram-positive cocci (2- to 4 fold) and Gram-negative bacilli (2- to 8 fold) (220, 222), an excellent oral bioavailability, a lower potential for drug interactions and a superior activity in biofilm (220).

PK/PD studies support once daily administration, whereas higher serum concentrations and better tolerability are some more advantages compared to its ancestor (220, 221, 223). In a phase 1 study of healthy volunteers, eravacycline achieved in the ELF concentrations greater than plasma by 6-fold and in the alveolar macrophages by 50-fold, indicating the potential for treatment of MDR bacteria causing pneumonia (224).

*In vitro* studies of eravacycline against *E. coli* resistant to 3rd generation cephalosporines and fluoroquinolones showed a potent *in vitro* activity with an MIC90 of 0.5 mg/L (221, 222). A recent *in vitro* study compared eravacycline and commonly used antibiotics against a collection of 284 CRAB isolates possessing an acquired OXA or a metallo-beta-lactamase, or expressing an up-regulated intrinsic OXA-51-like enzyme (225). Eravacycline was demonstrated as the most potent *in vitro* agent vs. all comparators (reference beta- lactams, aminoglycosides, colistin, tetracyclines, sulbactam and fluoroquinolones) displaying the lowest MIC_50/90_ values of 0.5/1 mg/L, respectively. Colistin resistance in this collection was 13.3% with relevant MIC_50/90_ values of 1/4 mg/L, respectively. No correlation was found between _bla_OXA genes and eravacycline MICs, indicating an excellent candidate in the treatment of CRAB infections.

IGNITE1 and IGNITE4 (Investigating Gram-negative Infections Treated with Eravacycline) were randomized, double-blind, double-dummy, multi-center Phase 3 clinical trials in cIAIs, in which eravacycline administered at a dose of 1 mg/kg IV q12h was compared to ertapenem (1 g IV every 24 h) and meropenem (1 g IV every 8 h), respectively. In both studies non-inferiority criteria were met (226, 227); adverse events were low for a drug of this class, with nausea, vomiting, and diarrhea reported at 5, 4, and 3%, respectively (227). Based on these data eravacycline was approved for cIAIs by both FDA and EMA in 2018 (120, 121).

IGNITE 2 and 3 were phase 3 randomized, multi-center, double-blind, clinical trials in cUTIs, in which eravacycline administered at a dose of 1.5 mg/kg IV once daily was compared to levofloxacin (750 mg every 24 h) and ertapenem (1 g every 24 h), respectively. Non-inferiority criteria were not met and UTI indication was withdrawn (122). Although failure may be attributed to the lower dose employed in cUTIs compared to the cIAI trial, there are additional concerns about clinical efficacy of tetracycline class in UTIs.

In light of the available *in vitro* and *in vivo* data, eravacycline holds promise as a key player in the treatment of infections caused by *A. baumannii* being the first launched antibiotic with potent activity against this notorious pathogen since decades. Real life data will determine its real impact on patients severely ill and with multiple co-morbidities. Keeping in mind the paradigm of tigecycline for which data from healthy volunteers did not translate successfully in critically ill patients, and the failure of eravacycline in pivotal RCTs in UTIs, caution is required. When this new drug will enter clinical practice, surveillance for resistance and PK/PD studies in special populations (i. e. mechanically ventilated patients or on CRRT), are of utmost importance to ensure optimal patient outcomes (70, 71).

## How to Integrate New and Old Antibiotics in Clinical Practice

In this comprehensive review we tried to appraise currently available treatments for MDR and XDR pathogens. Our ultimate aim was to provide hints on how to integrate recently marketed antibiotics in the challenges of clinical practice, particularly referring to CRE, MDR *P. aeruginosa* and CRAB. It is of great importance to emphasize that the novel beta-lactam/beta-lactamase inhibitor combinations, such as those containing avibactam and vaborbactam, are promising to be the best options for the treatment of CRE where carbapenem resistance is mediated by KPC. *In vitro* all these inhibitors are active against Enterobacteriaceae producing ESBL, KPC-2, KPC-3, and AmpC, whereas only avibactam inhibits certain class D β-lactamases, mainly OXA-48. Although they have been subjected to a rigorous program of development, their “real life” efficacy against CRE remains unascertained because clinical trials design were not focused on the treatment of infections caused by XDR and PDR pathogens (115). As far as CAZ-AVI is concerned, an almost 3-years in-market experience has brought in light some important studies comparing its efficacy vs. colistin. The first study employing a prospective design, highlighted the probability of better outcome of 64% compared to colistin (193), whereas a second retrospective study demonstrated CAZ-AVI as the only variable associated with survival (192). These data indicate that CAZ-AVI may represent the most appropriate so far choice of CRE mediated by KPC or OXA-48. Further to the activity against OXA enzymes, CAZ-AVI's important antipseudomonal activity is an advantage for empirical regimens in high risk patients and in mixt infections. The addition of aztreonam, still marketed individually, could expand CAZ-AVI's spectrum to MBL-producers (228, 229). On the other hand, meropenem-vaborbactam seems to have a lesser potential for selection of resistance among KPC-producers than CAZ-AVI (230, 231), while demonstrating similar efficacy against KPC-2 and KPC-3 (203). In addition, meropenem possesses excellent anti-anaerobe coverage and can stand alone in the treatment of intraabdominal infections without the addition of metronidazole. Fortunately, so far, no cross resistance is reported between these two antibiotic combinations (203). The compilation of real-life data for meropenem-vaborbactam will enable us to understand better its true position in the treatment of CRE infections.

The disadvantage to be seriously considered by the clinician is the inability of both CAZ-AVI and meropenem-vaborbactam to inhibit metallo-β-lactamases (MBLs) i.e., VIM, IMP, and NDM, as well as *A. baumannii* (115). The knowledge of the epidemiology of CRE infections in each country is of major importance. OXA-48 like enzymes are more prevalent in France, Spain and Belgium, whereas NDM-1 is most frequently isolated in Southeast Asia countries and India, whereas regional or inter-regional spread of NDM in Europe has been reported in Belgium, Denmark, France, Romania, Poland, Turkey, and Greece (11). In terms of empiric therapy of CRE infections, taking into consideration the epidemiological profile of each region, agents active against both serine β-lactamases and MBLs are indicated. The predominance of KPC could indicate the administration of either CAZ-AVI or meropenem-vaborbactam. However, in case of the emergence of MBLs the combination with colistin, should be considered to cover both possibilities. Figure 2 provides an algorithm for optimal integration of new and old antibiotics in the treatment of CRE.

It is evident that combination empiric regimens are mandatory in areas with MDR prevalence. The big question, is whether new antibiotics against CRE would abide the need of combination in definitive treatments, for which data from observational studies are clearly in favor, being contradicted by data from meta-analyses (8, 25, 26, 45, 232, 233). Factors elucidated as independent predictors of mortality or treatment failure of CAZ-AVI treatment are septic shock, neutropenia, high comorbidity scores, recent ventilation, pneumonia, and receipt of renal replacement therapy outlining a critical role for combination with another active *in vitro* antibiotic in these cases (190, 192, 193). However, the latter position is arbitrary and based mostly on experience before the new beta-lactamase combinations requiring targeted and prospective randomized trials. Figure 1 summarizes conditions that must be met in order to decide monotherapy in the treatment of MDR pathogens, based on the existing literature and expert driven recommendations (8, 26, 57, 58, 95, 234).

When *P. aeruginosa* is considered, combination treatment is the rule in the empirical phase (58). In definite treatment, data from meta-analyses found no benefit for combination treatments, however clinicians hardly accept monotherapy in patients with major risk factors for mortality, in bacteraemic infections and septic shock (233, 234). The launching of ceftolozane-tazobactam expands beta-lactam activity in CR strains, providing a potent backbone; colistin, fosfomycin and plazomicin could act as companion agents (Figure 3). CAZ-AVI as well may contribute as a backbone antipseudomonal agent in the new era (161).

When CRAB is prevalent, colistin or tigecycline are the only currently available treatments (69). Launching of eravacycline holds promise, however real-life data from CRAB infections and critically ill patients are lacking. Other newer agents against CRAB i.e., cefiderocol are welcomed and high clinical cure rates are expected (7, 9). Regarding CRAB resistant to colistin, one unanticipated finding from the exploratory subgroup analysis of the AIDA study revealed lower mortality among patients with colistin-resistant isolates when treated with colistin monotherapy over combination of colistin -meropenem, with a possible explanation being the loss of fitness and virulence relative to colistin-susceptible strains (232). Indeed, cumulative data have shown that combination treatments against *A. baumanni* might be less necessary compared to CRE, to ensure optimal outcomes (233, 234).

In summary, the novel combinations are not to be considered “panacea” for the ongoing crisis in the therapy of XDR Gram-negative bacteria and colistin is still considered as possessing a fundamental position for the treatment of CRE in combination (particularly in areas where MBL predominate) as well as for the treatment of CRPA (in many cases being the only *in vitro* active drug) as well as CRAB. CAZ-AVI and meropenem-vaborbactam can be used as backbones in the treatment of CRE and under circumstances, they could be used as definitive monotherapy. Similarly, ceftolozane-tazobactam could be seen as an ideal beta-lactam backbone for the treatment of MDR *P. aeruginosa*, as well as a stand-alone antibiotic, when monotherapy is sought and conditions fulfilled. Plazomicin could absorb some volume of colistin prescriptions, both in empiric and definitive treatment of difficult-to-treat pathogens, subject to local epidemiological evidence. Finally, fosfomycin as a companion antibiotic for *P. aeruginosa* and CRE infections, deserves a greater attention, as it has no cross resistance with other antibiotic classes, may retain susceptibility against a variety of resistance mechanisms to other antibiotics and possesses negligible toxicity. Lessons learnt from the pauci-antibiotic era, make vigilance for emergence of resistance a priority. Furthermore, it is important to minimize use of these new precious antibiotics as empiric treatments and focus on microbiologic documentation of infections; this will ensure their longevity in our armamentarium.

## Author Contributions

GP: conception, writing, and supervising of the manuscript. IK, SL, KP, and VR: literature review, writing parts of the manuscript. All authors have approved the final version.

### Conflict of Interest Statement

GP has received speaker's honoraria and/or grants from Angelini, MSD, Pfizer. KP has received speaker's honoraria from MSD and Pfizer. The remaining authors declare that the research was conducted in the absence of any commercial or financial relationships that could be construed as a potential conflict of interest.
